# A review of ancestrality and admixture in Latin America and the caribbean focusing on native American and African descendant populations

**DOI:** 10.3389/fgene.2023.1091269

**Published:** 2023-01-19

**Authors:** Thais C. De Oliveira, Rodrigo Secolin, Iscia Lopes-Cendes

**Affiliations:** ^1^ Department of Translational Medicine, School of Medical Sciences, University of Campinas (UNICAMP), Campinas, Brazil; ^2^ The Brazilian Institute of Neuroscience and Neurotechnology (BRAINN), Campinas, Brazil

**Keywords:** vulnerable population, admixture, population genomics, global health, global South

## Abstract

Genomics can reveal essential features about the demographic evolution of a population that may not be apparent from historical elements. In recent years, there has been a significant increase in the number of studies applying genomic epidemiological approaches to understand the genetic structure and diversity of human populations in the context of demographic history and for implementing precision medicine. These efforts have traditionally been applied predominantly to populations of European origin. More recently, initiatives in the United States and Africa are including more diverse populations, establishing new horizons for research in human populations with African and/or Native ancestries. Still, even in the most recent projects, the under-representation of genomic data from Latin America and the Caribbean (LAC) is remarkable. In addition, because the region presents the most recent global miscegenation, genomics data from LAC may add relevant information to understand population admixture better. Admixture in LAC started during the colonial period, in the 15th century, with intense miscegenation between European settlers, mainly from Portugal and Spain, with local indigenous and sub-Saharan Africans brought through the slave trade. Since, there are descendants of formerly enslaved and Native American populations in the LAC territory; they are considered vulnerable populations because of their history and current living conditions. In this context, studying LAC Native American and African descendant populations is important for several reasons. First, studying human populations from different origins makes it possible to understand the diversity of the human genome better. Second, it also has an immediate application to these populations, such as empowering communities with the knowledge of their ancestral origins. Furthermore, because knowledge of the population genomic structure is an essential requirement for implementing genomic medicine and precision health practices, population genomics studies may ensure that these communities have access to genomic information for risk assessment, prevention, and the delivery of optimized treatment; thus, helping to reduce inequalities in the Western Hemisphere. Hoping to set the stage for future studies, we review different aspects related to genetic and genomic research in vulnerable populations from LAC countries.

## Introduction

The use of human genomic data has grown in recent decades, generating unique knowledge about the biology of different populations (1000 Genomes Project Consortium et al., 2015; Mallick et al., 2016; Karczewski et al., 2020). Also, in recent years there has been a better appreciation of the importance of genomic variability in human health and disease, leading to an increase in worldwide initiatives aiming to obtain in-depth information about population genomics in the context of precision medicine (PM) (Aronson and Rehm, 2015; Hindorff et al., 2018). However, traditionally large-scale studies include predominantly individuals of European and Asian ancestry, and the benefits derived from these studies have been mainly focused on high-income countries^1^ (1000 Genomes Project Consortium et al., 2015; Turnbull et al., 2018; Martin et al., 2019). More recently there is an increase in genetic and genomic studies in the Global South, where most populations have different degrees of admixture including African, Native American, and European.

Advances in the genomics of human populations have demonstrated that Africa harbors the greatest genetic variation and diversity compared to other continents (Tishkoff et al., 2009; 1000 Genomes 20151000 Genomes Project Consortium et al., 2015; Gurdasani et al., 2015). The genetic variation found in Eurasia, Oceania, and the Americas is largely a subset of sub-Saharan African diversity (Ramachandran et al., 2005; Jakobsson et al., 2008; Schlebusch et al., 2012), with small contributions from archaic non-sub-Saharan African humans (Green et al., 2010; Meyer et al., 2012; Prüfer et al., 2014). Thus, it is a consensus that population genomic studies in African populations are crucial to advance the knowledge about human genomic diversity. Fortunately, the Human Heredity and Health in Africa (H3Africa) consortium aims to close some gaps in the lack of sub-Saharan African genomes, with at least 70,000 participants across different ethnolinguistic groups, including whole-genome sequencing and genotyping data as well as an association with phenotype cohorts (Mulder et al., 2018). Even though prominent projects with European ancestry and new consortia such as H3Africa continue to increase our understanding of global genetic variation (Genome of the Netherlands Consortium, 2014; 1000 Genomes 20151000 Genomes Project Consortium et al., 2015; Yamaguchi-Kabata et al., 2015; Fakhro et al., 2016), most association studies still focus on simple admixture scenarios (Martin et al., 2019; Wojcik et al., 2019). Hence, little is known about the role of genetic diversity in admixed populations.

The Americas harbors the largest number of countries with admixed ancestries; however, only recently admixed Americans started to be included in population genomics studies. Indeed, the first substantial genomic study based on the USA and directed to its admixed populations was opened for enrollment in 2018 under the name “All of Us Research Program’, which aims to sequence more than 1 million individuals from different ethnic backgrounds found in the United States. In 2019, 175,000 participants were included in the program, where 75% were from groups historically underrepresented in biomedical research and 45% were from racial and ethnic minorities (All of Us Research Program Investigators et al., 2019; Mapes et al., 2020). Nonetheless, Latin America and the Caribbean (LAC), which comprises Central and South America and the Caribbean islands, is still severely underrepresented in genetic studies due to insufficient large-scale publicly available data (Ledda et al., 2014).

Since LAC is composed of population groups considered vulnerable due to historical oppression and territorial usurpation^2^, such as individuals of Native Americans and sub-Saharan African background, it is essential to be aware of specific legal and ethical aspects involving genomic research in these vulnerable populations. These include questions related to the sovereignty of the data collected. In this scenario, we present a review of ancestry in the context of genomic access to global health in admixed LAC populations. We also aim to verify the ethical and social issues created by the current demand for genomic data and how genomics associated with ancestry can be applied to public health and PM for the benefit of vulnerable admixed populations of LAC countries.

## History before and after colonization in LAC

LAC is the cultural definition of a region encompassing Central America (Belize, Costa Rica, El Salvador, Guatemala, Honduras, Mexico, Nicaragua, and Panama), South America (Argentina, Bolivia, Brazil, Chile, Colombia, Ecuador, Guiana, French Guiana, Paraguay, Peru, Suriname, Uruguay, and Venezuela), and the Caribbean islands (Antigua and Barbuda, Aruba, Bahamas, Barbados, Cuba, Dominican Republic, Granada, Guadalupe, Haiti, Cayman Islands, Turks and Caicos Islands, Virgin Islands, Jamaica, Marticinica, Puerto Rico, Saint Barthelemy, Santa Lucia, Sao Cristovao e Neves, Sao Vicente and Granadinas, and Trinidad Tobago). According to the Population Divisions of the Department of Economic and Social Affairs from the United Nations (UN), in 2021 the total population in LAC had reached 656,098,000^3^. These people present a rich admixture of languages and cultures, which have helped them to shape distinct identities for centuries (Sánchez-Albornoz, 1974; Reich et al., 2012). Despite the diversity of people and cultures throughout LAC, they have generally been formed mainly by the admixture from three different continent populations: Native Americans (America continent), Europeans (Europe), and sub-Saharan Africans (Africa) (Adhikari et al., 2016). This is relevant considering the diversity of Native American peoples throughout the Americas and the diversity of African individuals that contribute to the great diversity found in black communities in the Americas nowadays.

Peopling of LAC began from the Northeast Asian population, who crossed Beringia and split between a North Native American branch and a Central/South Native American branch about 17,000–13,000 years ago (Nielsen et al., 2017; Mendes et al., 2020). They have adapted to different geological conditions and ecological characteristics throughout the Central/South American continent, creating at the same time genetic diversity and a unique genomic structure in the Late Pleistocene (Chacón-Duque et al., 2018; Pinotti et al., 2019). Currently, they are Maya, Nahua, Aymara, and Quechua individuals (Mao et al., 2007); some Peruvian individuals (1000 Genomes Project Consortium et al., 2015; Mallick et al., 2016); and Brazilian indigenous populations (Mas-Sandoval et al., 2019; Castro e Silva et al., 2020). Indeed, studies have shown a correlation between the spatial pattern of genetic diversity with geography, environment, and linguistic and cultural diversity among Native American peoples, where populations of western South America appear more homogeneous compared with those of eastern South America (Tarazona-Santos et al., 2001; Barbieri et al., 2019; Borda et al., 2020).

The consequence of this human dispersion and adaptation was the rise of the great pre-Columbian civilizations in the Mesoamerican region, including the Mayas, Incas, Aztecs, and Teotihuacanos (Roca-Rada et al., 2020), and the Tupi ethnic groups found on the Brazilian coast and French Guiana in the 15th century (Mazières et al., 2007; Mazieres et al., 2011; Castro e Silva et al., 2020).

Interestingly, signs of natural selection have also been reported among Native Americans for several genes related to lipid metabolism, body development (Amorim et al., 2015), high soil concentration of arsenic in the Atacama Desert (Vicuña et al., 2019), high altitude in the Andes (Tarazona-Santos et al., 2000), and the immune system (Hsueh et al., 2018; Borda et al., 2020; Ávila-Arcos et al., 2020). Indeed, human skeletal analysis revealed that diseases such as treponematosis and tuberculosis were already present in LAC, but with a low prevalence in some groups (Larsen, 1994; Diamond, 1997; O’Fallon and Fehren-Schmitz, 2011).

However, the social and biological scenario of Central/South Native Americans dramatically changed with the mass migration of Europeans in the 15th century and the sub-Saharan African diaspora from the 16th to 19th centuries (Ferro, 1997; Bergad, 2001). The Spanish and Portuguese were the two main populations from Europe who dispersed across all of LAC from the 15th century on. Since then, conflicts have occurred against Native Americans and several Native American populations were enslaved into forced hard labor (Wade, 2010). Indeed, even after Native American slavery had been outlawed in Spanish colonies in 1542 and Portuguese colonies in 1570, several peripheral regions maintained those practices (Wade, 2010). However, different from the relative early protection laws about enslavement of Native Americans, the concept of slavery and individual freedom of sub-Saharan Africans received little questioning until centuries later. Thus, between the 16th and 19th centuries, an estimated 12.5 million sub-Saharan Africans were brought to Spanish and mainly Portuguese colonies (Eltis et al., 2007; Heywood and Thornton, 2007) to work on sugar cane, coffee, tobacco, cotton, and cacao plantations, in addition to gold and silver mining (Wade, 2010). It is important to note that some population heterogeneity occurred during the transatlantic slave trade (TAST), where the Caribbean islands received most of the individuals from West Africa. On the other hand, Brazil and Rio de La Plata region received mostly individuals from West-Central and Southeast Africa, with 84% of enslaved people just from Angola, according with the Transatlantic Slave Trade Database^4^. This historical dispersal also reflects the sub-Saharan African ancestry among admixed populations from LAC (Table 1) (1000 Genomes Project Consortium et al., 2015). In addition, several enslaved sub-Saharan Africans escaped and formed resistant settlements called *maroons* or *palenques* from the Spanish, and *quilombos* in Brazil (Wade, 2010; Nunes et al., 2016).

**TABLE 1:** Global ancestry proportions among Latin America and the Caribbean countries.

Country	Subpopulation/Sample location	N	NAT	EUR (%)	AFR (%)	References
Bahamas	General population	221	NA^d^	4.0	96.0	Simms et al. (2010)
Cuba	Diabetes Care Center of Havana, with nationwide coverage	229	1.0%	73.0	26.0	Diaz-Horta et al. (2010)
	Individuals representing all provinces	1,019	8.0%	72.0	20.0	Marcheco-Teruel et al. (2014)
	Voluntary participants representing all provinces	860	8.0%	71.0	21.0	Fortes-Lima et al. (2018)
Jamaica	General population	119	NA^d^	16.0	78.3	Simms et al. (2010)
	General population	44	8.0%	10.0	82.0	Torres et al. (2013)
	Jamaican Adolescent Asthma Study	45	1.0%	11.0	89.0	Mathias et al. (2016)
Haiti	General population	111	NA^d^	4.0	96.0	Simms et al. (2010)
St. Thomas	General population	99	5.6%	16.9	77.4	Torres et al. (2013)
St. Kitts	General population	47	5.8%	8.2	85.9	Torres et al. (2013)
Dominican Republic	Genes-environments and Admixture in Latino Asthmatics study	47	9.0%	52.0	38.0	Mathias et al. (2016)
Puerto Rico	Genes-environments and Admixture in Latino Asthmatics study	53	12.0%	61.0	27.0	Mathias et al. (2016)
	Puerto Ricans from Puerto Rico^e^	104	12.9%	73.2	13.9	Martin et al. (2017)
Dominica	General population	37	16.0%	28.0	56.0	Torres et al. (2013)
St. Lucia	General population	50	7.0%	18.0	75.0	Torres et al. (2013)
Barbados	General population	39	NA^d^	16.0	84.0	Mathias et al. (2016)
	African Caribbeans in Barbados^e^	96	0.3%	11.7	88.0	Martin et al. (2017)
St. Vincent	General population	51	6.0%	13.0	81.0	Torres et al. (2013)
Grenada	General population	48	7.0%	12.0	81.0	Torres et al. (2013)
Trinidad and Tobago	General population	43	9.0%	16.0	75.0	Torres et al. (2013)
Mexico	Mexico City, States of Guerrero, Chihuahua, Jalisco, Puebla, Yucatan, Sonora, Nuevo León, Zacatecas, Guanajuato, Hidalgo, Veracruz, Campeche	NA	62.0%	31.0	6.0	Moura et al. (2015)
	Mexican Ancestry from LA, USA^e^	64	47.0%	48.7	4.3	Martin et al. (2017)
Guatemala	Oriente	20	53.0%	40.0	7.0	Wang et al. (2008)
Honduras	Garífuna population	41	17.0%	2.0	81.0	Mathias et al. (2016)
Nicaragua	Departments of Chinandega, Leon, Managua, Carazo, Chontales, Matagalpa, Esteli, Madriz, Nueva Segovia, Jinotega, Atlántico Norte, and Atlántico Sur	165	11.0%	69.0	20.0	Nuñez et al. (2010)
Costa Rica	Central Valle of Costa Rica	20	28.7%	66.7	4.6	Wang et al. (2008)
	Central Valle of Costa Rica	3,996	38.0%	58.0	4.0	Ruiz-Narváez et al. (2010)
Panama	Provinces of Cocle, Colon, Chiriquí, Herrera, Los Santos, Panama, and Veraguas	4,202	35.9%	25.4	38.7	Arias et al. (2002)
	Provinces of Cocle, Colon, Chiriquí, Herrera, Los Santos, Panama, and Veraguas	800	51.0%	25.0	24.0	Castro-Pérez et al. (2016)
Venezuela	Caracas, States of Falcón, and Zulia	NA	25.0%	60.0	14.0	Moura et al. (2015)
Colombia	Medellin, Departments of Nariño (Pasto), Antioquia (Peque), and Cundinamarca	78	44.0%	42.0	11.0	Wang et al. (2008)
	Caribbean Coast, Southwest Andean Region, Vale del Cauca, Colombian mountain range of Los Andes, and populations settled in the Amazonian Region and Oriental flats	NA	38.1%	48.7	13.2	Godinho et al. (2008)
	Colombians from Medellin, Colombia^e^	94	27.4%	62.5	9.2	Homburger et al. (2015)
	Colombians from Medellin, Colombia^e^	94	25.7%	66.6	7.8	Martin et al. (2017)
	Medellin and Chocó	723	29.7%	49.0	21.3	Chande et al. (2021) ^a^
Ecuador	NA	19	50.1%	40.8	6.8	Homburger et al. (2015)
Peru	25 localities from Andean, Amazonian, and Coastal regions	551	92.0%	6.0	2.0	Moura et al. (2015)
	Lima	64	68.3%	26.0	3.2	Homburger et al. (2015)
	Peruvians from Lima, Peru^e^	85	77.3%	20.2	2.5	Martin et al. (2017)
Brazil	38 different populations from all five geographic regions	8,733	17.0%	62.0	21.0	Moura et al. (2015)
	Northeast (Salvador), Southeast (Bambuí), and South (Pelotas)	6,487	7.0%	65.8	27.1	Kehdy et al. (2015) ^b^
	Individuals predominantly from Southeastern region	264	7.0%	76.9	13.8	Secolin et al. (2019)
	Elderly people recruited from São Paulo city	1,171	6.7%	72.6	17.8	Naslavsky et al. (2022)
Bolivia	Andean region (La Paz), and Sub-Andean region (Chuquisaca)	178	81.0%	17	2.0	Heinz et al. (2013)
	Departments of Beni, Chuquisaca, Cochabamba, La Paz, Pando, and Santa Cruz, Chimane, Moseten, Aymara, Quechua, Ayoreo, Trinitario, Yuracare, Ignaciano, and Movima	720	71.0%	25.0	2.0	Taboada-Echalar et al. (2013)
Paraguay	Asunción, and departments of Cordillera, Caaguazú, Paraguari, Misiones, Guairá, Itapúa, Alto Paraná, Amambay	548	33.8%	55.4	10.8	Simão et al. (2021)
Uruguay	NA	NA	10.4%	84.1	5.6	Hidalgo et al. (2005)
	Native Americans with Charruá, Guaraní/Charruá, and Guenoa ancestries	10	22.2%	68.6	9.1	Spangenberg et al. (2021)
Argentina	Salta, Tucuman, and Catamarca^c^	52	48.7%	47.7	3.6	Wang et al. (2008)
	Pampa, Gran Chaco, Mesopotamia, Northwest region, and Patagonia	NA	38.0%	58.9	3.1	Godinho et al. (2008)
	Provinces of Buenos Aires, Corrientes, Formosa, Chaco, Misiones, and Jujuy	NA	42.0%	54.0	3.0	Moura et al. (2015)
	Multiple sites in Buenos Aires, Cordoba, Mar del Plata, Rosario, Santa Fe, and Mendoza	175	27.7%	67.3	3.6	Homburger et al. (2015)
Chile	Paposo, Quetalmahue	40	56.0%	42.0	2.0	Wang et al. (2008)
	Santiago	27	38.7%	57.2	2.5	Homburger et al. (2015)
	Metropolitan region, Arica y Parinacota, Tarapacá, Antofagasta, Atacama, Coquimbo, Valparaíso, O’Higgins, Maule, Bio-bio, Araucanía, Los Ríos, Los Lagos, Aysén, and Magallanes	313	43.2%	54.4	2.4	Eyheramendy et al. (2015)

Notes: The sum of the proportions for some data is not 100% because we extracted only the three populations that have shaped Latin America and the Caribbean (based on the literature). Global ancestry was inferred by ADMIXTURE, ADMIXMAP, and RFMix software. The genetic markers include the ABO blood system, ancestry informative markers (AIMs), short tandem repeats, microsatellites, single nucleotide polymorphisms (SNPs), SNP arrays, and whole-genome sequencing datasets.

^a^Weighted average of the two populations evaluated in the study.

^b^ Global ancestries were based on the mean of the three Brazilian cities analyzed in the study.

^c^ Global ancestries were based on the mean of the three locations analyzed in the study.

^d^ In this study the proportions were estimated for AFR, EUR, and East Asians ancestry.

^e^ Populations from the 1000 Genomes Project.

AFR, african ancestry; EUR, european ancestry; NAT, native american ancestry; NA, data not available.

Besides warfare and slavery, there is a consensus that the population sizes of Native Americans decreased rapidly after contact with Europeans. Diseases that had not been present in the Americas until European and sub-Saharan African contact played a crucial role in that population decrease, including bubonic plague, measles, smallpox, mumps, chickenpox, influenza, cholera, diphtheria, typhus, and leprosy (Larsen, 1994; Diamond, 1997; O’Fallon and Fehren-Schmitz, 2011). In addition, the encouragement of European colonization of LAC and the arrival of sub-Saharan African populations by TAST led to an admixture and greater fragmentation of Native American genomes (Adhikari et al., 2016; Pena et al., 2020), which has impacted the way that we handle diverse diseases and phenotypes in LAC populations. Thus, leading to current discrepancies in the quality of health care provided to these populations in LAC, with black and indigenous people having less access to health services and care, increasing the prevalence of many diseases in these groups. Also, most health-related studies in LAC are normally centered in patients of European background, causing a general lack of knowledge about the health conditions and prevalent diseases in the underprivileged communities of native and afro-descendants.

Noteworthy is the additional challenge in reconstructing indigenous ancestral origins, primarily due to the lack of data on ancestral indigenous populations that dates back to the genocide of these peoples in the beginning of European colonization. A similar scenario may also be seen for the chromosomes derived from the African descendants in LAC; however, using genome-wide ancestry of descendants of 17th-century enslaved Africans, it was possible to trace the origin of African descendants to Bantu-speaking groups from north Cameroon and non-Bantu-speaking groups from Nigeria and Ghana. This was the first published paper to provide evidence of the ethnic origins of Africans in LAC (Schroeder et al., 2015). African populations are genetically variable, and descendant populations have a dynamic admixture associated with drift patterns, geographic isolation over time, and even different migration patterns (Moreno-Estrada et al., 2013; Mathias et al., 2016; Martin et al., 2017). However, the dynamic population interactions remain incomplete, with several gaps in genetic information about West-Central Africa, from where the largest source of enslaved Africans came (5.7 million people) (Eltis et al., 2007). Genotyping and genomic population studies across the entire American continent and West-Central African countries (Angola, Congo, and Sierra Leone, and people who speak Kehoe-San) have helped to obtain a more accurate picture of the contribution of the three significant ancestries (Europe, Africa, and Native Americans) and to quantify the genetic impact of the TAST (Micheletti et al., 2020). In that work the authors report that the African ancestry in the Americas is consistent with historical data of TAST, with the most recent common ancestors and highest values of pairwise identity-by-descent (IBD) alleles shared between the American and West-Central African populations. Also, using millions of individuals from different databases (including 23andMe’s ancestry inference algorithm), it was found that Latin America harbors less sub-Saharan African ancestry compared with North America (8.9%–19.9% compared with 45%–76%), and British Caribbean and the Guianas (76% and 59%, respectively), with different proportions of ancestries from the four African groups (Nigerian, Senegambian, Coastal West Africa, and Congolese). Even though, the study could be biased in the estimations for North America, since it focused only on data from individuals from the coastal regions of the United States, the information of a greater African ancestry in North America, when compared with LAC is indeed curious since more than 70% of the African enslaved contingent came to LAC. However, the findings by Micheletti et al. (2020) suggest a lower effective population and higher mortality in the group of enslaved African populations that came to LAC (Bergard, 2007; Browning et al., 2018). Further studies are important to clarify these findings.

Adding to the complexity of population admixture in LAC, other European and Asian populations also arrived in South America, mainly after the politics of slavery prohibition in the late 19th and early 20th centuries, adding more structure to the ancestry patterns of these populations. Italians, Germans, Dutch, British, Ukrainians, Slavs, Russians, Japanese, and even immigrants from the United States and Canada have settled mainly in French Guiana, Southeast/South Brazil, Uruguay, Paraguay, and Argentina (Alonso, 2007; Mazieres et al., 2011; Pena et al., 2020).

## Admixture in LAC

Studies in ancestry and admixture in LAC are growing in the last decade, even though the region is still underrepresented in the biomedical literature, especially when considering the Caribbean countries. Although genomes from LAC populations are a mosaic of the three ancestral populations, the proportion of each of them differs significantly among countries and even among subpopulations within each country (Kehdy et al., 2015; Moura et al., 2015; Adhikari et al., 2016; Secolin et al., 2019; Naslavsky et al., 2022). Table 1 shows the proportions of Native American, European, and sub-Saharan African ancestry among LAC countries, based on various genetic markers, from ABO blood system, short tandem repeats (STRs), microsatellites, and insertions or deletions (InDels), to high-density single nucleotide polymorphism (SNP) dataset and whole-genome sequencing. However, when analyzing the data presented in Table 1 one should be aware of ascertainment bias in the genetic and genomic studies in LAC populations. This is due mainly to the great variability present within countries, making it difficult to generalize the results obtained from populations of a specific geographic region to the entire county. All countries encompassing LAC present a level of admixture from the three ancestral populations, except for Haiti and Barbados showing only European and sub-Saharan African admixture. Of note, ancestry feasibly reflects the demographic history of LAC: Caribbean islands present a higher proportion of sub-Saharan African ancestry, as demonstrated by studies from Bahamas, Cuba, Jamaica, Haiti, Dominican Republic, Puerto Rico, Dominica, St. Lucia, Barbados, St. Vincent, Grenada, and Trinidad and Tobago (Benn-Torres et al., 2008; Bryc et al., 2010; Diaz-Horta et al., 2010; Simms et al., 2010; Torres et al., 2013; Marcheco-Teruel et al., 2014; Mathias et al., 2016; Martin et al., 2017; Fortes-Lima et al., 2018); Central America, Andean regions, and Ecuador have populations with high Native American ancestry (Arias et al., 2002; Wang et al., 2008; Nuñez et al., 2010; Ruiz-Narváez et al., 2010; Moura et al., 2015; Castro-Pérez et al., 2016; Mathias et al., 2016; Martin et al., 2017); and Brazil, Paraguay, Uruguay, Argentina, and Chile present high proportions of European ancestry (Ruiz-Linares et al., 2014; Homburger et al., 2015; Moura et al., 2015). Although based on mtDNA and Y-chromosome length polymorphisms, Native American subpopulations from French Guiana also present a proportion of Sub-Saharan African ancestry, which derived from historical contact with a group of escaped slaves who lives in the region nowadays (Mazières et al., 2007). Noteworthy, a special case has occurred in El Salvador, where historical events of human rights violations and the aftermath 1980–1992 civil war have produced many unidentified casualties and missing individuals. These events, lead to a group of parents of missing children, organized in the Pro-Búsqueda Association^5^, to generate a database of genetic profiles of relatives and young people already found. Based on forensic markers, this initiative has revealed that individuals in El Salvador are closer to the US Hispanics from autosomal STRs, whereas X-STRs markers were found closer to Native American populations (Casals et al., 2022).

Furthermore, a close look at the LAC countries also reveals the different distribution of ancestry proportions in subpopulations and regions, as observed in Cuba (Fortes-Lima et al., 2018), Garifuna population in Honduras (Herrera-Paz et al., 2010), Mexico (Rubi-Castellanos et al., 2009; Silva-Zolezzi et al., 2009), Venezuela (Moura et al., 2015), Colombia (Godinho et al., 2008; Ibarra et al., 2014; Mogollón Olivares et al., 2020; Chande et al., 2021), Bolivia (Heinz et al., 2013; Taboada-Echalar et al., 2013), Paraguay (Simão et al., 2021), Argentina (Godinho et al., 2008), Chile (Eyheramendy et al., 2015) and Brazil (Kehdy et al., 2015; Moura et al., 2015; Mychaleckyj et al., 2017; Secolin et al., 2019; 2021). Also, as observed in several studies, the admixture events were sex biased and occurred between European males and Native American and/or sub-Saharan African females in Cuba (Mendizabal et al., 2008), Jamaica (Deason et al., 2012), Dominican Republic (D’Atanasio et al., 2020), Nicaragua (Nuñez et al., 2010), Belize (Monsalve and Hagelberg, 1997), El Salvador (Lovo-Gómez et al., 2007; Casals et al., 2022), Panama (Migliore et al., 2021), Costa Rica (Carvajal-Carmona et al., 2003), Mexico (Green et al., 2000), Ecuador (González-Andrade et al., 2007), Colombia (Carvajal-Carmona et al., 2003), Brazil (Pena et al., 2011), Uruguay (Sans et al., 2002), and Argentina (Dipierri et al., 1998). In addition, mitochondrial DNA (mtDNA) and Y chromosome data have shown that European ancestry comes mainly from the male parent, while sub-Saharan African ancestry comes from the female parent in Brazil (Alves-Silva et al., 2000; Silva et al., 2006; Manta et al., 2013), Bolivia (Taboada-Echalar et al., 2013), and Argentina (Salas et al., 2008). These findings indicate non-consensual sexual harassment of enslaved women, a phenomenon known to occur frequently during colonization (Baptist, 2001; Bergard, 2007; Malareck, 2011). This sex bias is remarkable in LAC compared with the Americas colonized by the British, where for every African man, 4 to 17 African women contributed to the gene pool (Micheletti et al., 2020).

Understanding genomic and ancestry information has already led to the development of some PM initiatives in LAC. For example, the major histocompatibility complex (MHC) has been strongly selected for African ancestry in Mexican individuals (Zhou et al., 2016). In the Dominican Republic, a study revealed that women patients with obesity and type 2 diabetes presented a higher proportion of sub-Saharan African ancestry in comparison with women without obesity and type 2 diabetes (Tajima et al., 2004). In Chile, ancestry was inferred from high-density SNP genotype data of 313 individuals from two case-control studies examining hantavirus infection and 22q11 microdeletion syndrome in residents of the country (Eyheramendy et al., 2015). In Colombia, ancestry analysis among 624 individuals from Medellín and 99 individuals from Chocó revealed higher correlations between ancestry and disease prevalence risk by polygenic risk score (PRS) estimates (Chande et al., 2021). In Peru, the Peruvian Genome Project (Harris et al., 2018) and the 12G and 100G-MX Projects (Romero-Hidalgo et al., 2017; Aguilar-Ordoñez et al., 2021) generated whole-genome data from Native American populations. Also, a recent preprint study from “The Mexico City Prospective Study” analyzed high-density SNP genotype data and whole-exome/whole-genome sequencing of over 140,000 adult Mexicans and correlated it to some phenotypic traits (Ziyatdinov et al., 2022). The data was partially from whole genome sequencing (WGS) and whole exome sequencing (WES) with majority of genome-wide genotyping, and a new imputation reference panel based on WGS was developed to access common variants with high proportion of Native ancestry in Mexico (Ziyatdinov et al., 2022). Furthermore, a study demonstrated that including Native American genomes improved the representation of rare variants and the imputation performance of LAC genomes (Jiménez-Kaufmann et al., 2022). Most recently, the project JAGUAR launched in 2021 (Joining all: Genes, immUnity And diveRsity) will map immune cells across Latin America to create the first high-resolution genetic atlas across different ancestries, to determine the impact of ancestry in the immune system^6^.

Similarly, to other admixed American populations, the Brazilian population is derived from sub-Saharan Africa, Europe, and Native American populations (Kehdy et al., 2015; Moura et al., 2015; Mychaleckyj et al., 2017; Secolin et al., 2019; 2021). Although it is underrepresented in the global context of genomic studies, in the last 10 years projects such as 1) *DNA do Brasil* (Patrinos et al., 2020), 2) the Brazilian Initiative on Precision Medicine (BIPMed) (Rocha et al., 2020), 3) the Health, Welfare, and Aging Project (SABE) (Naslavsky et al., 2022), and 4) EPIGEN-Brasil (Lima-Costa et al., 2015) have attempted to understand and map the genomes of the Brazilian population. DNA do Brasil proposed sequencing more than 15,000 individuals to understand the phenotypic impact of genetic variations in the Brazilian population and the genetic contribution to disease susceptibility through the construction of Native American and sub-Saharan African genomes (Patrinos et al., 2020). The initiative began in 2020 but was discontinued in 2022. Fortunately, the researchers started a start-up biotech Gen-t which aims to sequencing 200,000 genomes from different regions of the country to contribute to PM in Brazil^7^. The largest genomic study published on the Brazilian population based on whole-genome sequencing, which includes 1,171 elderly people in the city of São Paulo (Southeast), showed that approximately 2 million variants are absent from large public databases. Therefore, it is very likely that these variants that are associated with genes have been inherited from populations also underrepresented in the references, such as Native Americans and sub-Saharan Africans (Naslavsky et al., 2022). Projects from BIPMED have reported similar data based on SNP arrays and whole-exome sequencing. Specifically, there was a decreased proportion of European ancestry along with an excess of Native American ancestry on chromosome 8p23.1, a region containing genes associated with obesity, type 2 diabetes, lipid levels, and waist circumference (Secolin et al., 2019; 2021). Furthermore, different genomic regions could influence traits based on ancestry. Indeed, one study from 958 children of the Social Change, Asthma, Allergy in Latin America (SCAALA) Cohort in Salvador, Brazil, showed that African ancestry at the 17q21.31, 10q22.2, and 2p23.1 *loci* was associated with lower lung function. In contrast, the European ancestry at the 17q21.31 locus showed the opposite effect (Fonseca et al., 2020).

Together with Latin American researchers, the International Common Disease Alliance (ICDA)^8^ has strived to generate a Latin American cohort to provide genomic and ancestry information at subpopulation and country levels from LAC. The importance of these data goes far beyond understanding LAC’s demographic history. Indeed, it is known that the main clinical phenotypes associated with metabolic disorders among admixed Americans include type 2 diabetes, insulin secretion, body mass index, obesity, and adiposity (Dunn et al., 2006; Hayes et al., 2013; Goetz et al., 2014; Flores et al., 2016). Studies have shown that PRS estimated from genome-wide association studies (GWASs) based on European populations may not be informative for admixed American individuals, even with a significant proportion of European ancestry (Martin et al., 2017; 2019). For example, some variants associated with generalized genetic epilepsy found in European-based GWAS did not replicate in admixed Brazilian patients, and the shreds of evidence pointed to different ancestral backgrounds in admixed Brazilian patients (Kaibara et al., 2021). Indeed, guidance published by the American Society of Human Genetics emphasizes the development of diverse research cohorts, including underrepresented populations (Novembre et al., 2022). Therefore, compared with other populations, LAC populations need a self-genomic reference to estimate PRS more accurately and to provide better personalized treatment.

## Vulnerable remnant populations

Communities of Native Americans and sub-Saharan African descendants (Maroons/Quilombolas) are considered socially vulnerable populations in LAC, not only due to the historical events but also the continuing suffering from racism both at the individual level and from the structures, including governmental entities that should protect their rights and needs. Mostly, they are still struggling to be recognized as traditional communities and to access basic human rights (Luna, 2006). These communities are underrepresented in the economic and political scenarios and suffer from disparity in the health care system and difficulties accessing them (Naciones Unidas - Comisión Económica para América Latina y el Caribe (CEPAL), 2018). It is important to point out that Native Americans and sub-Saharan Africans had different aspects of enslavement during colonization. Africans were brought to be enslaved for 3 centuries and the Native Americans at some point were decimated. The few rights acquired in recent years are unequally distributed to these two populations compared to the populations of each country. For example, in Colombia, as much as indigenous people have suffered and still suffer from discrimination and abuse, in 2005, they owned 30% of the territory legally acquired in the form of reserves, although they only represented 3% of the total population. Sub-Saharan African descendants have been ignored and marginalized before the law, with academic studies about them beginning only in the 1990s (Wade, 2010). In Brazil, at least under the Brazilian Constitution of 1988, many rural Quilombola communities could have land rights based on Afro-Brazilian ancestry and settlement (Gomes, 2003; French, 2006).

On social and economic issues, both groups are indicators of poverty or extreme poverty for people living in rural and urban areas in LAC countries with data (Brazil, Ecuador, Panama, and Peru), according with CEPAL^9^ and the World Bank Group^10^. Unfortunately, indigenous peoples represent the lower strata of the economic chain, having little or no representation in the upper-middle- and high-income strata, and are more affected in urban areas. As a result, in Brazil the inequality rate is more than two times greater in sub-Saharan African descendants and indigenous peoples compared to the general population. Furthermore, these groups can still experience xenophobia and discrimination regarding their origin, ethnicity, or race when they attempt international migration (United Nations - Economic Commission for Latin America and the Caribbean (ECLAC), 2019).

The lack of genetic and genomic studies about indigenous peoples and Maroons/Quilombolas in LAC may also impact better understanding of the health and socio-economic conditions of these populations. For example, studies show that indigenous populations have a higher infant mortality rate than non-indigenous populations, with examples ranging from 19/1,000 in Colombia, 22/1,000 in Ecuador, 99/1,000 in Peru, and 106/1,000 in Brazil (Montenegro and Stephens, 2006). Furthermore, high mortality rates can have a devastating effect on small indigenous populations and can contribute to the end of an entire population (Napolitano and Stephens, 2003). Moreover, the intense contact with other populations due to economic activities, such as mining exploration in Argentina, has revealed high concentrations of heavy metals in the hair of native populations (Cabrera, 2003).

The issues in access to the health system and the transition from an agricultural to a more industrialized food source make indigenous populations more susceptible to complex diseases such as high blood pressure, obesity, and anemia (Angeli et al., 2011; Freitas et al., 2011; Kimura et al., 2012; Mujica-Coopman et al., 2015; Popkin and Reardon, 2018). Other diseases as sexually transmitted infections (Mckenna, 1993; Dias et al., 2021); and infectious diseases such as malaria, yellow fever^11^, intestinal parasites, and coronavirus disease 2019 (COVID-19) are also a concern for theses populations (Flowers, 1994; Beltrame et al., 2002; Montenegro and Stephens, 2006; Hotez et al., 2008; Damazio et al., 2013; Moreira et al., 2022). So far, approximately 625 million people throughout the world have been infected by severe acute respiratory coronavirus 2 (SARS-CoV-2), the cause of the COVID-19 pandemic^12^, of which almost 12% are in Latin America^13^. In addition, populations that lack access to quality health care, such as Native Americans and Maroon/Quilombolas, have been significantly affected by infectious diseases, including COVID-19. Indeed, these populations offered a public health challenge due to the inaccurate reports of COVID-19 infection (Johansson et al., 2021), difficulties in accessing their territories and poor socio-economic conditions. In the specific case of Brazil, in which the Quilombolas and indigenous groups were prioritized during the COVID-19 vaccination, the acquisition of vaccines by the government had a considerable delay, which affected the entire Brazilian population, and the difficulties to deliver the vaccines mainly to locations with access only possible by boat, interfered with the maintenance of vaccine’s refrigeration^14^. These issues also led to low vaccination rates in these communities (Moreira et al., 2022).

Problems with access to services (including health care) and insufficient democratization of their rights are still a sad daily routine. In 2003, the Unit of Indigenous Communities and Community Development reviewed 21 LAC countries according to variables of best legislative practices. Among the countries with the best rankings are Argentina, Ecuador, Nicaragua, and Panama, followed by Brazil, Colombia, and Venezuela (Zamudio, 2003). One of the categories of legal rights was whether the country had special legislation for the health of natives and whether it included special access to the local health system, according with the Indigenous and Tribal Peoples Convention, 1989^15^. In addition, after the requests of the 2021 UN conference, Brazil and Colombia have attempted to promote adequate access to education, new technologies, and the legal system for black people, under policies designated to promote “racial equality” (Gonçalves, 2011).

## Native American communities and their genomic history

**FIGURE 1:**
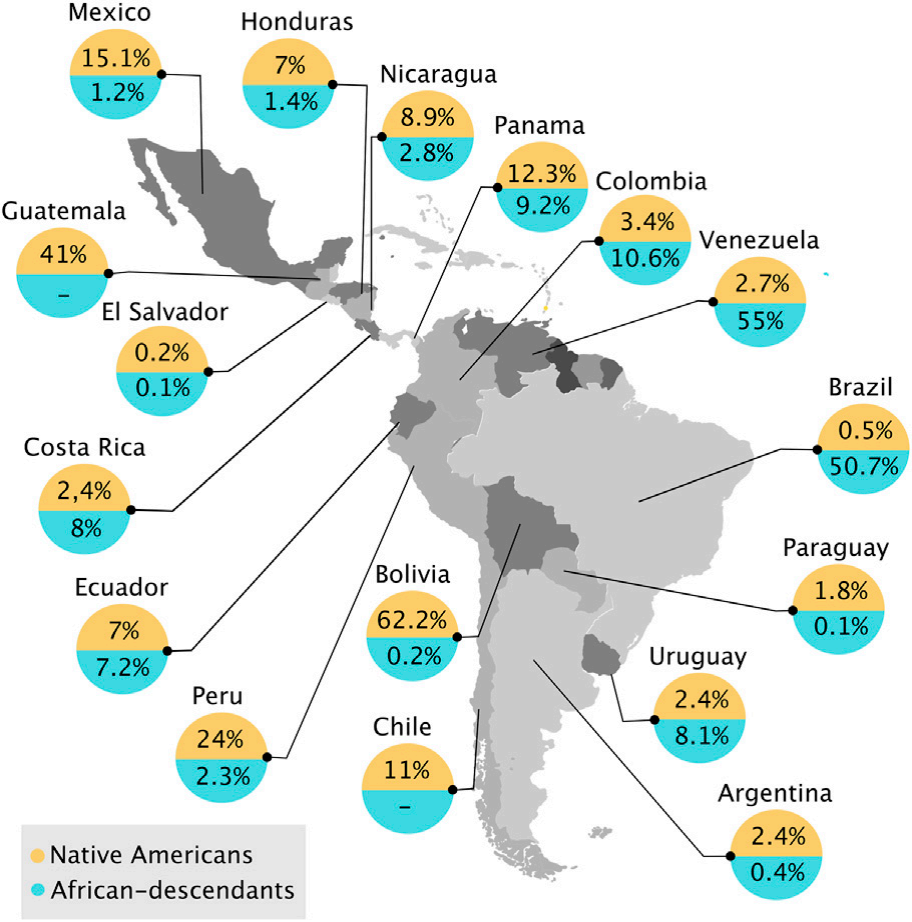
Indigenous and sub-Saharan African descendant populations in Latin America and the Caribbean in 21st century. (-) represents a lack of data. Map generated from open-source R software and populations values adapted from https://repositorio.cepal.org/bitstream/handle/11362/39964/S1600174_en.pdf?sequence=5&isAllowed=y.

It is estimated that 52.4–150 million indigenous people already inhabited LAC before the arrival of Europeans in the 15th century (Thornton, 1987; Denevan, 1992). Unfortunately, the majority of these populations and ethnicities became extinct during the colonization process (Thornton, 1987; Stannard, 1993). In 2010, over 800 Native American groups lived in LAC, speaking more than 1,000 languages and dialects, which comprises 8.3% of the total population of LAC^16^ (Valeggia, 2016). Guatemala (41%), Panamá (13.3%), and Mexico (15.1%) have the highest percentage of indigenous peoples in Central America (Figure 1). It has been demonstrated the predominance of Native American genetic contribution (54%–69%) in 641 individuals from Native American and Mestizo groups in Guatemala, El Salvador, Nicaragua, and Panama (Baeta et al., 2021). In Guatemala (in which 41% are indigenous people) a study showed that Guatemala-Native Americans are highly similar to the Mayan populations in Mexico (Martinez-Gonzalez et al., 2016). The Amerindian populations in Nicaragua (8.9%) and Costa Rica (2.4%) showed a high degree of paternal genetic differentiation, even though they share geographic proximity (Melton et al., 2013). In South America, Bolivia stands out^17^, as this country has predominant Native American features: 62.2% of the population self-identifies as Native American (Figure 1), and only 0.5%–12% of people have European ancestry (Galanter et al., 2012; Watkins et al., 2012; Taboada-Echalar et al., 2013). In Ecuador, which harbors 7% of Native Americans, a study showed that Mestizos had predominant Native American (Kichwas) (71.2%) ancestry compared with European contribution (Poulsen et al., 2011). Brazil, where only 0.8 million people are self-declared Native Americans, harbors the greatest number of different indigenous backgrounds, more than three times compared with Colombia, the country with the second most different indigenous backgrounds. It also has the most indigenous people in danger of physical or cultural disappearance^18^. South America itself includes three domains of Native American communities: the Pacific Coast, the Andes, and Amazonia. The three have experienced different cultural and social effects, ranging from expansive complex societies found by Incas to collectors in the Amazon Rainforest (Tarazona-Santos et al., 2001; Fuselli et al., 2003; Dixon and Aikhenvald, 2006; Barbieri et al., 2014). The coast and the Andes populations share a similar ancestry and population size. At the same time, the Amazon region has high levels of consanguinity, which could reflect the small isolated model proposed by previous study (Tarazona-Santos et al., 2001). This model could perhaps work for almost all isolated Amazonian populations, but there are some exceptions. Amazonian populations display long-distance sharing of large and short fragments with the Andes and the Coast and a non-uniform history, which is not expected for small population sizes (Barbieri et al., 2019).

The study of Native Americans currently found in LAC is of particular interest because the mixture involving them took place locally—that is, the indigenous ancestral genomic that is part of contemporary indigenous peoples is consistent with the same region they occupied in the past. This could lead to a better understanding of genomic structures and genetic diversity of pre-Columbian Native American populations (Castro e Silva et al., 2020). At the same time, colonization appears to be less sensitive to indigenous populations in the Andes (Harris et al., 2018) and the Amazon Rainforest (Castro e Silva et al., 2022), which were the last territories to be colonized and also are the ones that were not fully colonized, due to the geography of the territories. Therefore, the genetic history of Native Americans is challenging due to the lack of availability of complete genomes from ancestral and current populations. The study of the Native American component, as found in admixed Brazilian populations (on average 7%), can be challenging (Kehdy et al., 2015; Secolin et al., 2019). Despite several difficulties, efforts have also been made to analyze high-density SNP data and/or whole-exome/whole-genome sequencing from Native American groups. In Uruguay, researchers performed whole-genome sequencing of 10 Uruguayan individuals with self-declared Charruan heritage. The resulting haplotypes were enriched in these Charruan descendants and are rare in the rest of the Native American groups evaluated (Spangenberg et al., 2021). In Peru, clotting factor genes were associated with preeclampsia in pregnant women living in Puno and the nearby Peruvian Andes, a high-altitude region (Nieves-Colón et al., 2022). Interestingly, the results also showed an average of 97.5% Native American ancestry in the sample, which is not present within the Native American ancestry among Peruvians from Lima and Mexicans (1000 Genomes Project Consortium et al., 2015). This ancestry seems to derive from the Andean highland-specific Native American ancestry found in previous studies (Harris et al., 2018; Barbieri et al., 2019; Borda et al., 2020). In Brazil, one study sequenced the exomes of 58 Native Americans from the east Amazon region, and the results strengthened the genetic distinction between Andean and Amazonian populations (Castro e Silva et al., 2022). Although, the most promising study at LAC on indigenous people were in Mexico: genome-wide analysis from 716 individuals from 60 diverse ethnic groups showed strong influence by geography inside the country, and low divergence between other Natives from North and South America, when compared with other worldwide populations (García-Ortiz et al., 2021).

Even though there is a scarcity of data, studies have tried to recreate a portion of the history of Native Americans that has been obscured by European colonization, generating data that complement the history of both these populations and the evolution of their genomes (Castro e Silva et al., 2020). A recent study of Native American populations in Brazil resulted in the reconstruction of individuals that emulate the ancestral native populations (Mas-Sandoval et al., 2019). Although the contribution of this study to corroborate the hypothesis that there is a genetic reservoir of Native American diversity in admixed populations in Brazil is limited (due to the lack of ancient DNA), they authors found evidence of a decrease diversity in non-admixed current Native American populations, in comparison to the reconstructed populations ancestral native genomes (Mas-Sandoval et al., 2019). The genetic reconstruction of Native American populations that no longer exist as non-mixed populations indicates the possibility of recovering lost chapters of LAC history and may reveal, at least genetically, the constitution of the peoples who lived in this region before the arrival of the Europeans.

The distribution of new, rare alleles and clinically relevant variations among indigenous individuals remain largely unknown, negatively affecting the understanding of genetic contributions to Mendelian and complex diseases affecting these populations and the current descendants (Popejoy and Fullerton, 2016; Landry et al., 2018). Therefore, there is a need for a complete genome of adequate reference in contrast to those of European descent (1000 Genomes Project Consortium et al., 2015; Turnbull et al., 2018; Nurk et al., 2022) and methodologies to analyze the complex miscegenation. The generation of new genomic data and the structure of diverse LAC populations would allow the identification of diseases and increase the accuracy of risk scores, leading to the possibility of implementing the discoveries in genomic medicine to LAC populations (Swart et al., 2020). Conducting population genomics studies in indigenous LAC populations is essential to form the foundations of genomic medicine that could be implemented in these countries. Therefore, Native American genomes and portions of indigenous DNA that remain in the current admixed populations are expected to show genetic alterations that originated in current or even extinct Amerindian populations. In addition, it may help to understand genetic variants found in the current LAC populations.

## The communities of enslaved sub-Saharan African descendants: The maroons/quilombolas

The Maroon/Quilombola communities stand out among the economically disadvantaged groups, self-declared black and brown people who commonly face inequalities regarding illness and death (Batista et al., 2004; Cardoso et al., 2005; Lopes, 2005; Volochko and Vidal, 2010). The definition of Maroon/Quilombolas is based on its historical trajectory and presumption in black ancestry related to resistance to the historical oppression suffered between the 16th and 19th centuries, during the colonial era, by individuals who escaped slavery, abandoned individuals, or freed, formerly enslaved people (Price, 1996; Nunes et al., 2016). These communities persisted in the Americas, and the Noir Maroon is one of the largest populations, with relative isolation across the decades and the conservation of cultural traditions and language between South Guiana and Suriname (Price, 2002). Using millions of genotype markers between the Noir Maroon population, the Quilombolas in southeastern Brazil and Colombia, and populations that originated from Africa, Fortes-Lima et al. (2017) showed that the African-Brazilian and African-Colombian descendants have a lower proportion of African ancestry (74%–76%) than the Noir Maroon (97%). Indeed, both Noir Marron and African-Colombian populations are strongly associated with the Bight of Benin and the Gold Coast. At the same time, African-Brazilian individuals have affinities with West-Central Africa, which is consistent with the trafficking routes from different African countries to Central and South America (Fortes-Lima et al., 2017). Moreover, even if this population in South America does not promote consanguineous marriage, they lived in relative genetic isolation which helps to understand the high proportion of African origins in a scenario with long-term genetic isolation reflected in ROH estimates (Price and Price, 2003; Kirin et al., 2010). The gene pool characteristic that stands out in the Noir Maroon population is that the male ancestry originates from Senegal to Benin, and the female ancestry comes from the Ivory Coast to Angola, showing differential migration based on the sex of the individuals (Brucato et al., 2010).

In contrast, little attention has been paid to the African-Bolivian communities created under TAST. Although most of the Bolivian population is indigenous, these small communities have been neglected and have limited access to the larger cities; they have mainly remained isolated after the agrarian reform of 1952 (Lipski, 2006). mtDNA is a suitable tool for tracing contemporary “African-American” haplotypes. Although the ancestry of African-Bolivian individuals could not be traced using only mtDNA, the genetic influence of East and Southeast Africa can be partially explained by ancestry from the Middle East (Heinz et al., 2015). This region was one of the few that during the TAST received people from regions of Africa that overlapped with the Arab slave trade (Lovejoy, 2011).

Although there are still too few publications on genetic and genomic studies of the Maroon/Quilombola communities of LAC, there has been research in Brazil in the last decade. Currently, there are more than 5,970 remaining *quilombos* (areas protected by the government where these populations live; IBGE, 2010^19^), between the North and South regions, with a community concentration formed by 100–400 individuals in Southeast Brazil (Santos and Tatto, 2008). However, no more than 30 communities have been analyzed regarding their genomic background (Kimura et al., 2017). In general, Quilombola communities are deprived of treated water sources, garbage collection, and sewage (Galindo et al., 2012), which could expose the residents to harmful substances and mutagenic agents. A genotoxicity and mutagenicity analysis revealed evidence of genomic damage in Quilombola communities in the State of Goiás, Brazil (de Moraes Filho et al., 2020). This lack of basic sanitation also promotes the transmission of several diseases and fosters precarious health conditions in these communities, which have been considered one of the most vulnerable populations in Brazil (Tavares and Silva, 2014; Melo and Silva, 2015; Santos et al., 2020; Britodos et al., 2022). Interestingly, some of these communities are small and because the practice of consanguineous marriages was normalized mainly during the first generations after their establishment, has led to a low degree of genetic diversity within communities, but a relatively large heterogeneity among different comunities (Lemes et al., 2018). In general, when compared with other world populations, Maroons showed a higher-than-average degree of gene flow but are similar to other African origins (Cotrim et al., 2004). The ancestrality of Quilombos communities in Southeast Brazil is commonly associated with the pattern of the three distinct populations, which are divided almost equally between sub-Saharan Africans and Europeans (approximately 39% each) and a smaller share by Native Americans (Kimura et al., 2013). Therefore, it is curious for an actual Brazilian population to have a >10% Amerindian contribution. In one study based on the *HLA-A*, *HLA-B*, *HLA-C*, and *HLA-DRB1* genes in Quilombolas from the Vale do Ribeira in Southeast Brazil, the authors inferred 150 *HLA* haplotypes from 133 *HLA* alleles. Among these 150 haplotypes, the author found 70 haplotypes in the National Marrow Donor Program (NMDP) public database, which ranks the haplotypes according to the “race/ethnic description” status. In this case, 29 haplotypes (41.4%) were ranked as African, 27 (38.6%) as European, and 14 (20.0%) (Nunes et al., 2016). These HLA genes are associated with several infectious diseases, including malaria (Hill et al., 1991) and COVID-19 (Dobrijević et al., 2022). In some Quilombolas of Northwest and North Brazil, we have a possible repository of Amerindian alleles, checked by informative ancestral markers with Quilombolas and other African-Brazilian populations, with over 40% Amerindian ancestry contributing to the admixture of Brazilian populations (Gontijo et al., 2014). Thus, it contains valuable information for studies in American pre-colonization history.

## The barriers to bioethics and data sovereignty

Bioethics, defined by critical and systematic reflections on ethical issues in medical research, biology, and public health (Luna, 2006), has been widely debated throughout the world when associated with minority populations at risk in terms of health, socio-economic, or cultural conditions^20^ ^,^ ^21^. Unlike North America and Europe, LAC only began the development of bioethics in the mid-1980s (Clotet, 2006). Currently, there are more than 700 publications in this area related to Brazil, Colombia, and Chile (Garcia et al., 2019). Ethics committees (ECs) began to develop in the early 1990s, but they are unequal and heterogeneous, and many countries still do not regulate their existence. Moreover, some of these ECs were created under the scope of contract research organizations, not guaranteeing the independence of these committees, and some were even created without having a relationship with research centers, which could generate a biased evaluation^22^. In LAC, at least three models of bioethics have arisen, but protection bioethics is the current one that evokes conflicts regarding health problems, especially in vulnerable populations (Schramm and Kottow, 2001; Schramm, 2008). The degree of vulnerability of the LAC countries and international regulations, such as the Declaration of Helsinki, determine that ECs have a major responsibility in protecting the human rights of research subjects^22^. The Universal Declaration on Bioethics and Human Rights (2005) by the United Nations Educational, Scientific and Cultural Organization (UNESCO) Member States established that ECs must access ethical issues related to projects involving human beings^23^.

Due to the historical events related to Native Americans and Maroon/Quilombolas and their vulnerabilities, there are broad requirements of ethical consent for research involving these groups. In LAC, the ethical process to collect any data, including socio-economic, demographic, phenotypic, and genotypic, is similar and involves several steps that begin with consent from the individual and the target community of the research, collected with the Free and Informed Consent Form (FICF) (Sequeiros et al., 2015). In Brazil, most of the ethical processes involving vulnerable populations are based not only in the agreement of the individual but also researchers must obtain the express agreement of the community involved and provide a detailed presentation of what will be done with these data. This process ensures equal contemplation of the interests involved, considering the vulnerability of the group in question and respecting the culture, religious belief, linguistics, and political and social structure ^24^ (Lopera, 2017). In 2005, Bolivia, with the highest percentage of the total population of Native Americans in LAC (Naciones Unidas - Comisión Económica para América Latina y el Caribe (CEPAL), 2014), reassured the vulnerability of the indigenous population in research topics based on several conventions and conferences, including the UNESCO recommendation concerning of scientific investigations of 1974^25^. In Brazil, where there are still indigenous communities partially or totally isolated from the rest of the population, there is a recommendation not to carry out research of any kind; for all the vulnerable groups, including Quilombolas, there is a recommendation that these groups are only studied if the investigation can bring benefits to them^26^. In Peru, where 2.3% of the population is considered to be of African ancestry^27^, if these communities cannot exercise their autonomy in consenting to research, measures could be taken to safeguard their rights^28^. Guatemala—the population of which is 41% indigenous^29^ has ensured that vulnerable people must be treated impartially and without any form of discrimination^30^. ECs in Chile (12.8% of the population self-identify as indigenous) have yet to develop specific protocols to include indigenous peoples in ethical sampling procedures that outline consent, privacy, interpretation of results, and scientific dissemination (Silva et al., 2022). Based on Resolution 1,498/2011^31^, the Ministry of Health in Argentina mentions ethnic minorities as vulnerable populations. However, the research proposal has no clear indication that Native Americans and African descendants are also included.

Unfortunately, despite evolving regulations for minorities, the low number of representative omics studies in these communities means there is still a lack of understanding of ancestry worldwide. For example, in databases such as the United Kingdom Biobank and the United States Health and Retirement Study, 77.5% of the data come exclusively from populations considered majority, that is, with European ancestry. The major explanation for the exclusion of minority groups is the confounding effect, which could lead to false evidence of causal variants, which is indeed a genuine concern (Ben-Eghan et al., 2020). Some studies have also claimed the lack of genetic population power in the analyses, generating possible statistically insignificant results (Ben-Eghan et al., 2020). However, omitting or not even looking for the data of vulnerable or minority populations leads to an ethical problem of under-representation and denial of the participation of these communities in PM. In this context, it is essential to understand the competing claims of genomic sovereignty when we define access to genomic data based on vulnerable populations. Considering indigenous populations, whose data are more sensitive than other populations (TallBear, 2013), the availability of indigenous genetic data may bring potential risk to these communities but little benefit (McInnes, 2011). Some North American indigenous communities have expressed a lack of confidence in the ability of ECs to ensure appropriate consultation (Garrison et al., 2019b) or to address unethical conduct (Woodbury et al., 2019). Some indigenous peoples have expressed concerns about the process of informed consent and access and benefit sharing of the generated data (Barelli, 2012; Hanna and Vanclay, 2013). Some American tribes understand the benefits of sharing data, but it is reflected in federal policies that do not allow for the oversight that tribal leaders need to carry out this responsibility (Garrison et al., 2019b; 2019a). Second, more genomic data and voice concerns are likely to be found in high-income countries than in low-/middle-income countries, as in LAC (Hudson et al., 2020). Lastly, “public trust, oversight, and long-lasting relationships with communities who participate in genomic research are required to advance both data sharing and diversity and inclusion—two major components of genomic research that must advance symbiotically for genomic research to benefit all” (Bentley et al., 2017).

In a review based on genomic access for indigenous populations in high-income countries, Hudson et al. (2020) showed that the current EC guidelines that would only arbitrate for access to genetic data are inconsistent with indigenous interests. Furthermore, the absence of representativeness for vulnerable populations in political and research consent organizations means that there is no one to advocate on their behalf regarding the use of their data. Therefore, three principles could lead to a better consideration of genomic data from Native groups.

(1) Building trust: Public access to genomic data and metadata must include the ability of these populations to access their data.
(2) Enhancing accountability: The data must be transparent, public, and disclosed in publications.
(3) Improving equity: Credit must be given to native populations to support any future research or use (Hudson et al., 2020).

We believe these principles should be considered in vulnerable populations, such as Maroons/Quilombolas and Native Americans from low-/middle-income countries.

An important example of a wrongdoing genetic access case happened in the 1960s with the Yanomami tribe located on the border of Venezuela and Brazil. The study was led by two American researchers who took blood samples without obtaining proper permission or following ethical procedures, and their actions impacted this tribe for decades. It took over 40 years for the reparation process to occur. The process of getting genetic and anthropological data from the Yanomami tribe was considered abusive, and the rights of the tribe were practically null, as the researchers did not obtain FICF. Moreover, the researchers did not provide health assistance after the study was completed. The greatest impact was the illegal collection of tribal members’ names and bartering to bribe the tribe to obtain critical cultural information (Guedes and Guimarães, 2020). Therefore, as well as having access to data, obtaining consent from the population being studied is critical. If the population is vulnerable in any respect, it is imperative to verify the best procedures for carrying out the study before, during, and after the entire process, evaluating how the results will impact the health, coexistence, culture, or socio-economic standards of that community.

## Conclusion

Knowledge about the structure of genomes from LAC populations, mainly Native Americans and Maroons/Quilombolas, is crucial for applying PM practices. It is also important to point out the current lack of medical genomic studies, including population ancestrality studies, especially from vulnerable populations from the Caribbean islands and Central American countries. Indeed, a critical goal of PM is that it should be inclusive and comprising information about the diversity of the human genome and how it interacts with environmental factors in the context of human health and disease. Therefore, genomics research on these vulnerable populations should include useful feedback regarding health, education, and quality of life for these communities. A more robust and comprehensive ethical process in research involving vulnerable populations compared with non-vulnerable populations should generate a positive chain of events in the process of getting the EC’s approval, which can last much longer than if these populations are not involved in the projects. Indeed, a thoughtful ethical process may help to ensure vulnerable populations who had been forced to submit and lose their identities during LAC colonization are not revictimized. Therefore, we believe that scientific policy development should empower vulnerable populations to participate in scientific research to ensure their genetic sovereignty and that they have a voice in the future use of data collected.

## Footnotes

1
https://databank.worldbank.org/data/download/site-content/CLASS.xlsx (Accessed 28 October2022).

2 World Bank Group, 2018. https://openknowledge.worldbank.org/handle/10986/30201 (Accessed 4 November 2022); United Nations - Economic Commission for Latin America and the Caribbean (ECLAC), 2019. https://www.cepal.org/en (Accessed 4 November 2022).

3
https://population.un.org/wpp/Download/Files/1_Indicators%20(Standard)/EXCEL_FILES/1_General/WPP2022_GEN_F01_DEMOGRAPHIC_INDICATORS_COMPACT_REV1.xlsx (Acessed 8 August 2022).

4
https://www.slavevoyages.org/voyage/database (Accessed 13 August 2022).

5
https://doi.org/10.1016/j.fsigen.2021.102646.

6
https://www.cambridgenetwork.co.uk/news/researchers-will-map-immune-cells-across-latin-america-support-czi (Accessed 4 November 2022).

7
https://gen-t.science (Acessed 4 November 2022).

8 ICDA. https://www.icda.bio/(Acessed 27 October 2022).

9
https://www.cepal.org/es/topics/demographic-projections/latin-america-and-caribbean-populationestimates-and-projections. (Acessed 8 August 2022).

10 World Bank Group, 2019. https://openknowledge.worldbank.org/handle/10986/30201 (Accessed 8 August2022).

11 Centro de Vigilância Epidemiológica “Alexandre Vranjac” da Secretaria de Saúde do Estado de São Paulo. Boletim Epidemiológico da Febre Amarela, 18 de fevereiro de 2019. http://www.saude.sp.gov.br/resources/cve-centro-de-vigilancia-epidemiologica/areas-de-vigilancia/doencas-de-transmissao-por-vetores-e-zoonoses/doc/famarela/2019/fa19_boletim_epid_1802.pdf (Accessed 8 August 2022).

12
https://covid19.who.int (Accessed 27 October 2022).

13
https://www.cambridgenetwork.co.uk/news/researchers-will-map-immune-cells-across-latin-america-support-czi (Accessed 4 November 2022).

14
https://apnews.com/article/brazil-coronavirus-pandemic-8f23692f52d83d9a2f1dc9fdffc34eaa (Accessed 13 August 2022).

15
https:// www.ilo.org/dyn/normlex/en/f?p=NORMLEXPUB:55:0::NO::P55_TYPE,P55_LANG,P55_DOCUMENT,P55_NODE:REV,en,C169,/Document (Accessed 30 August 2022).

16
https://www.geocities.ws/indiosbr_nicolai/summer/01.html (Accessed 28 October 2022).

17
https://www.cepal.org/en/infografias/los-pueblos-indigenas-en-america-latina (Accessed 30 August 2022).

18
https://www.cepal.org/en/infografias/los-pueblos-indigenas-en-america-latina (Accessed 30 August 2022).

19
https://www.ibge.gov.br/geociencias/organizacao-do-territorio/tipologias-do-territorio/27480-base-de-informacoes-sobre-os-povos-indigenas-e-quilombolas.html?=&t=acesso-ao-produto (Accessed 27 October 2022).

20
https://www.ohchr.org/es/special- procedures/sr-indigenous-peoples/international-standards-relating-indigenous-peoples (Accessed 30 August 2022).

21
https://salud.gob.ar/dels/entradas/etica-de-la-investigacion-en-salud (Accessed 30 August 2022).

22
https://salud.gob.ar/dels/entradas/etica-de-la-investigacion-en-salud (Accessed 30 August 2022).

23
https://e-legis-ar.msal.gov.ar/htdocs/legisalud/migration/html/5579.html (Accessed 27 October 2022).

24
https://www.gov.br/funai/pt-br/arquivos/conteudo/cogedi/pdf/legislacao-indigenista/pesquisa/resolucao-n-304-de-09-08-2000.pdf (Accessed 27 October 2022); https://clinregs.niaid.nih.gov/country/peru#participant_rights (Accessed 27 October 2022); https://salud.gob.ar/dels/entradas/etica-de-la-investigacion-en-salud (Accessed 27 October 2022).

25
https://www.lexivox.org/norms/INT-ACU-UNESCO31058.xhtml (Accessed 28 October 2022).

26
https://conselho.saude.gov.br/resolucoes/2012/Reso466.pdf (Accessed 28 October 2022).

27
https://www.worldbank.org/en/region/lac/brief/afro-descendants-in-latin-america (Accessed 27 October 2022).

28
https://www.gob.pe/institucion/presidencia/normas-legales/541080-011-2011-pcm (Accessed 27 October 2022).

29
https://www.censopoblacion.gt/mapas (Accessed 28 October 2022).

30
https://digi.usac.edu.gt/sitios/puiis/uploads/3/9/4/6/3946766/bioeticausac.pdf (Accessed 27 October 2022).

31
https://e-legis-ar.msal.gov.ar/ htdocs/legisalud/migration/html/18264.html (Accessed 27 October 2022).
