# Supplementary figures and images for: A review of ancestrality and admixture in Latin America and the caribbean focusing on native American and African descendant populations
Source: Front Genet. 2023 Jan 19;14:1091269. doi: 10.3389/fgene.2023.1091269 (PMC9893294; doi:10.3389/fgene.2023.1091269)

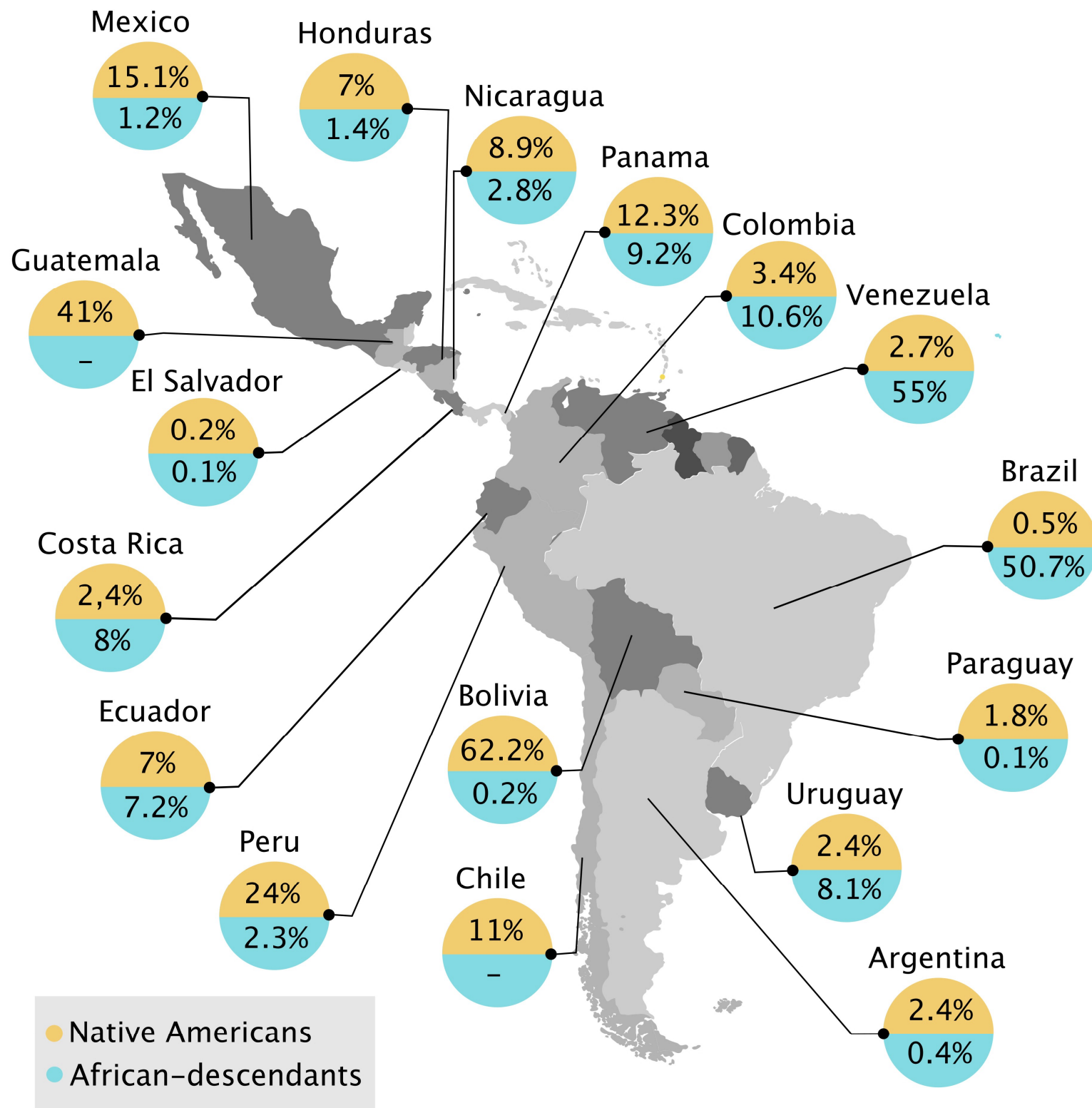

Supplement: Supplementary file 1 [file DataSheet1.PDF]
